# Follistatin‐like protein 5 inhibits hepatocellular carcinoma progression by inducing caspase‐dependent apoptosis and regulating Bcl‐2 family proteins

**DOI:** 10.1111/jcmm.13906

**Published:** 2018-09-25

**Authors:** Chunlei Li, Lei Dai, Junfeng Zhang, Yujing Zhang, Yi Lin, Lin Cheng, Hongwei Tian, Xin Zhang, Qingnan Wang, Qianmei Yang, Yuan Wang, Gang Shi, Fuyi Cheng, Xiaolan Su, Yang Yang, Shuang Zhang, Dechao Yu, Yuquan Wei, Hongxin Deng

**Affiliations:** ^1^ State Key Laboratory of Biotherapy and Cancer Center/Collaborative Innovation Center of Biotherapy West China Hospital Sichuan University Chengdu Sichuan China; ^2^ Department of Biochemistry Faculty of Basic Medicine Chongqing Three Gorges Medical College Wanzhou Chongqing China; ^3^ Institute of Immunology and Molecular Medicine Jining Medical University Jining Shandong China

**Keywords:** apoptosis, Bcl‐2 family proteins, caspase, FSTL5

## Abstract

Hepatocellular carcinoma (HCC) is one of the most common and deadly malignant tumors in the world, especially in China. Follistatin‐like protein 5 (FSTL5) is a member of the FSTL family, which is involved in cell proliferation, migration, differentiation, and embryo development. We aimed to investigate the function and underlying mechanism of FSTL5 in HCC. FSTL5 expression was determined by immunohistochemistry staining in a liver cancer tissue microarray (TMA) and the correlation between FSTL5 and the prognosis of HCC patients was analysed. Further proliferation assay, colony formation assay, flow cytometry, and xenograft tumor model were performed to investigate the bioeffects of FSTL5 in HCC in vitro and in vivo. We found that FSTL5 expression was downregulated in HCC tissues and positively correlated with the prognosis of patients with HCC at tumor node metastasis stage I/II. Overexpression of FSTL5 efficiently impaired HCC growth both in vivo and in vitro with an exogenous manner. Mechanistic investigation demonstrated that FSTL5 promoted HCC cell apoptosis in a caspase‐dependent manner and regulated Bcl‐2 family proteins. These results indicate that FSTL5 may be a potential novel target for HCC treatment, and a biomarker for tumor prognosis.

## 1 INTRODUCTION

Hepatocellular carcinoma (HCC) is the sixth most common cancer worldwide and the second most lethal cancer globally.^1^, ^2^ Surgical treatment is the most effective treatment for liver cancer, with a 5‐year survival rate of approximately 30%.^3^ The poor prognosis of patients with HCC is largely owing to the high frequency of tumor recurrence and distant metastasis after surgical resection.^4^, ^5^ The success of treating advanced HCC with sorafenib indicates that molecular targeted therapy is feasible for this malignancy.^6^ However, the therapeutic effects of this agent are far from satisfactory. Therefore, it is of great importance to find novel diagnostic biomarkers and therapeutic targets for HCC.

Follistatin‐like protein 1 (FSTL1, also known as FRP), FSTL3 (also known as FLRG), and FSTL5 compose the FSTL family.^7^, ^8^, ^9^ Although the function of FSTL family proteins is not fully understood, they seem to take part not only in regulating physiological processes, such as cell survival, proliferation, differentiation, and organ development, but also in regulating carcinogenesis and metastasis.^10^, ^11^, ^12^, ^13^, ^14^, ^15^ Hence, FSTL family members are considered attractive therapeutic targets and prognostic indicators.

FSTL5 was first discovered in adult and infant brain tissue in 1993, and it was partially sequenced.^9^ Not until 2002 was the complete FSTL5 gene sequence submitted to the Mammalian Gene Collection Program (MGC).^16^ FSTL5 is located on human chromosome 4q (mouse chromosome 3), and its 4867‐bp cDNA encodes a 93‐kD protein with 847 amino acid residues.^16^ FSTL5 was suggested to denote poor prognosis in medulloblastoma, which suggests an oncogenic role for this protein.^17^ Furthermore, Kim et al revealed that FSTL5 mutations are associated with resistance to XPO1 inhibitors in *KRAS*‐mutant lung cancer.^18^ Recently, Zender et al demonstrated that FSTL5 is a potential tumor suppressor gene in HCC by in vivo RNAi screening.^19^ Additionally, Zhang et al found that FSTL5 is downregulated and correlates with favourable prognosis in HCC.^20^ In this study, to further investigate the potential clinical significance, FSTL5 expression was determined by immunohistochemistry staining in a liver cancer tissue microarray (TMA) and the correlation between FSTL5 and the prognosis of HCC patients was analysed. Furthermore, we explored the biological effect and underlying mechanism of FSTL5 on HCC growth with proliferation assay, colony formation assay, flow cytometry, western blotting, and xenograft tumor model in vitro and in vivo. Our study would provide a novel therapy target and prognosis indicator for HCC.

## 2 MATERIALS AND METHODS

### 2.1 Clinical samples

Liver cancer TMA with survival data were purchased from Shanghai Outdo Biotech Co. Ltd. (Shanghai, China, HLiv‐HCC180Sur‐02, n = 180; HLiv‐HCC180Sur‐03, n = 180). Twenty‐six HCC tumor tissue samples with paired tumor tissue and normal tissue, which were confirmed histologically, were collected from HCC patients who underwent surgical resection at the West China Hospital from June 2009 to July 2013. Associated institutional ethics review board approved the use of these samples and the informed consents were obtained from all patients.

### 2.2 Immunohistochemistry

Tissue microarray was analysed for FSTL5 expression by immunohistochemistry (IHC) according to the manufacturer's recommendations (Vector Lab Inc., Burlingame, CA, USA). The antibody used is anti‐FSTL5 (Abnova, Taipei, TW, China).

Immunohistochemistry score was evaluated by two pathologists who were blinded to clinical information of the patients independently. The expression of FSTL5 was evaluated by Semi‐quantitative analysis. IHC was scored according to both the proportion of positively stained cells and the intensity of staining. The proportion of cells was scored as follows: 0 (<5%), 1 (5%‐25%), 2 (25%‐50%), 3 (50%‐75%), 4 (>75%). The intensity of staining was graded according to the following criteria: 1 (weak), 2 (moderate), 3 (strong). The final score was calculated as the product of the proportion of positive cells × the staining intensity score (range from 0 to 12) and was divided in 4 grades: ‐ (score of 0, 1 and 2), + (score of 3 and 4), ++ (score of 6 and 8) and +++ (score of 9 and 12).

### 2.3 RNA extraction and reverse transcription PCR

Total RNA from the cell lines and human tissues was extracted using Trizol (Invitrogen, Carlsbad, CA, USA). Total RNA was reverse transcribed into cDNA using RevertAid First Strand cDNA Synthesis Kit (Thermo Scientific, Waltham, MA, USA). PCR was performed according to the manufacturer's instructions. FSTL5 primers: forward 5′‐GCAGCAACTGTGGGACAAAG‐3′; reverse 5′‐CATGGCACCTAAGACTGGCA‐3′. β‐actin primers: forward 5′‐GGCATCGTGATGGACTCCG‐3′; reverse 5′‐GCTGGAAGGTGGACAGCGA‐3′.

### 2.4 Quantitative PCR and Western blotting

Quantitative PCR (qPCR) and western blotting were performed as described previously.^21^ FSTL5 primers: forward 5′‐AACAACTCACGCTTCAAG‐3′; reverse 5′‐TGTATGCTCCAGTATCTTCA‐3′. β‐actin primers: forward 5′‐TGGACTTCGAGCAAGAGATG‐3′; reverse 5′‐GAAGGAAGGCTGGAAGAGTG‐3′. The following primary antibodies were used in the western blotting assays:Anti‐FSTL5 antibody (1:1000; Sigma, St. Louis, MO, USA), β‐actin (1:5000; Santa Cruz Biotechnology, Santa Cruz, CA, USA), proliferating cell nuclear antigen (PCNA, 1:1000; Santa Cruz Biotechnology), Cleaved Caspase‐3 (1:1000; Cell Signaling, Boston, MA, USA), Cleaved Caspase‐8 (1:1000; Cell Signaling), Cleaved Caspase‐9 (1:1000; Cell Signaling), Bax (1:1000; Cell Signaling), Bad (1:1000; Cell Signaling), Puma (1:1000; Cell Signaling), P‐Bcl2(S70) (1:1000; Cell Signaling), P‐Bcl2(T56) (1:1000; Cell Signaling).

### 2.5 Xenograft tumors in nude mice

Studies were performed in accordance with institutional guidelines concerning animal use and care. Bel7404 and SMMC7721 cells (5 × 10^6^ cells) stablely expressing FSTL5 or control were injected subcutaneously into the flanks of female BLAB/c nude mice (5‐week old; Vital River Laboratories, Beijing, China). Tumor size was determined by collecting length and width with a sliding caliper every 3 days, and calculating the tumor volume (mm^3^) using the formula: length × (width)^2^ × 0.52. After mice were sacrificed, tumors from each animal were collected, weighed, and prepared for histopathological studies and western blotting.

### 2.6 Cell culture, transfection, plasmids, and lentivirus

HepG2, SNU398, SMMC7721, Bel7402, Bel7404, QGY7703, and SK‐Hep‐1 cell lines were obtained from the American Type Culture Collection (ATCC, Manassas, VA, USA). Cells were cultured in RPMI‐1640 (Gibco, Long Sheng Industry Park, Beijing, China) with 10% FBS (Gibco, Auckland, NZ, USA) with humidity at 37°C and 5% CO_2_. Cells were transfected by X‐tremeGENE HP DNA Transfection Reagent (Roche, Indianapolis, IN, USA). Plasmids pcDNA3.1(+), and pcDNA3.1(+)‐FSTL5 were purchased from GenScript (Nanjing, China). Lentivirus (LV) and Lentivirus‐FSTL5 (Lv‐FSTL5) were purchased from Hanheng (Shanghai, China). SiRNA targeting FSTL5 were purchased from Ribobio (Guangzhou, China) and used to transfect HCC cell line and LO2 cell following the instruction of ribo FECT ™ CP transfection kit (Ribobio, Guangzhou, China).

### 2.7 In vitro cell growth and colony formation assay

Cell growth was assessed with Cell Counting Kit‐8 (CCK‐8; Dojindo, Shanghai, China) according to the manufacturer's protocol. Transfected cells were seeded at 2 × 10^3^ cells per well in 96‐well plates. And then, absorbance was measured at 450 nm at different time points with microplate reader (Thermo Fisher scientific, Waltham, MA, USA).

Colony formation was assessed with crystal violet staining methods. Transfected cells were plated at 1000 cells per well into six‐well plates and cultured for approximately 14 days. And then, after crystal violet staining, cell number of each well was counted.

### 2.8 Flow cytometry assay for cell cycle and apoptosis

Cells were seeded onto a 6‐well plate and cultured.for cell cycle analysis, cells were harvested and fixed with 70% ethanol. The cell pellet was then treated with 50 lg/ml propidium iodide (PI; Sigma‐Aldrich, St. Louis, MO, USA) containing 0.1 mg/mL RNase A (Sigma‐Aldrich) and 0.1% Triton X (Sigma‐Aldrich), which was followed by flow cytometric analysis (BD Biosciences, San Jose, CA, USA). Cell apoptosis was measured with Annexin V‐Fluoresein isothiocyanate (Annexin V‐FITC) Apoptosis Detection Kit (KeyGEN, Nanjing, JS, China). Cells were collected and suspended in Binding Buffer. Then FITC Annexin V‐FITC and PI were added in sequence and mixed according to the manufacturer's instructions. After incubation in dark place, the stained cells were analysed by flow cytometry. A total of 30 000 events were analysed for each sample. Data were analysed using FlowJo 9.1 software (Tree Star Inc., Ashland, OR, USA).

### 2.9 Caspase‐3 activity assay

Caspase‐3 activity was measured by determining the level of chromophore *p*‐nitroaniline (pNA) after cleavage from the labelled substrate acetyl‐Asp‐Glu‐Val‐Asp *p*‐nitroanilide (Ac‐DEVD‐*p*NA) using Caspase‐3 Activity Assay Kit (Beyotime, Shanghai, China) according to the manufacturer's protocols. Collected cells were lysed with the lysis buffer followed by centrifugation (16 000 *g* for 15 minutes at 4°C). After that, the supernatant was transferred to pre‐cooled centrifuge tube. Caspase‐3 activity was assessed following the proteolytic cleavage of Ac‐DEVD‐pNA. Samples absorbance were measured at 405 nm.

### 2.10 Statistical analysis

Statistical analysis was performed using the SPSS Statistics software package (standard version 22.0; IBM, Armonk, NY, USA). The differences in categorical variables were analysed by chi‐square test or Fisher's exact test. The differences in continuous variables were analysed by Student's *t* test or one‐way analysis. The relationship between FSTL5 expression and clinicopathological characteristics of HCC patients was analysed by chi‐squared test. Survival curves were estimated by the Kaplan–Meier method and compared the difference by log‐rank test. The Cox proportional hazards regression model was used to identify the independent prognostic factors. A *P*‐value <0.05 was considered to indicate statistical significance.

## 3 RESULTS

### 3.1 FSTL5 expression is downregulated in HCC tissues and cell lines

**Figure 1:**
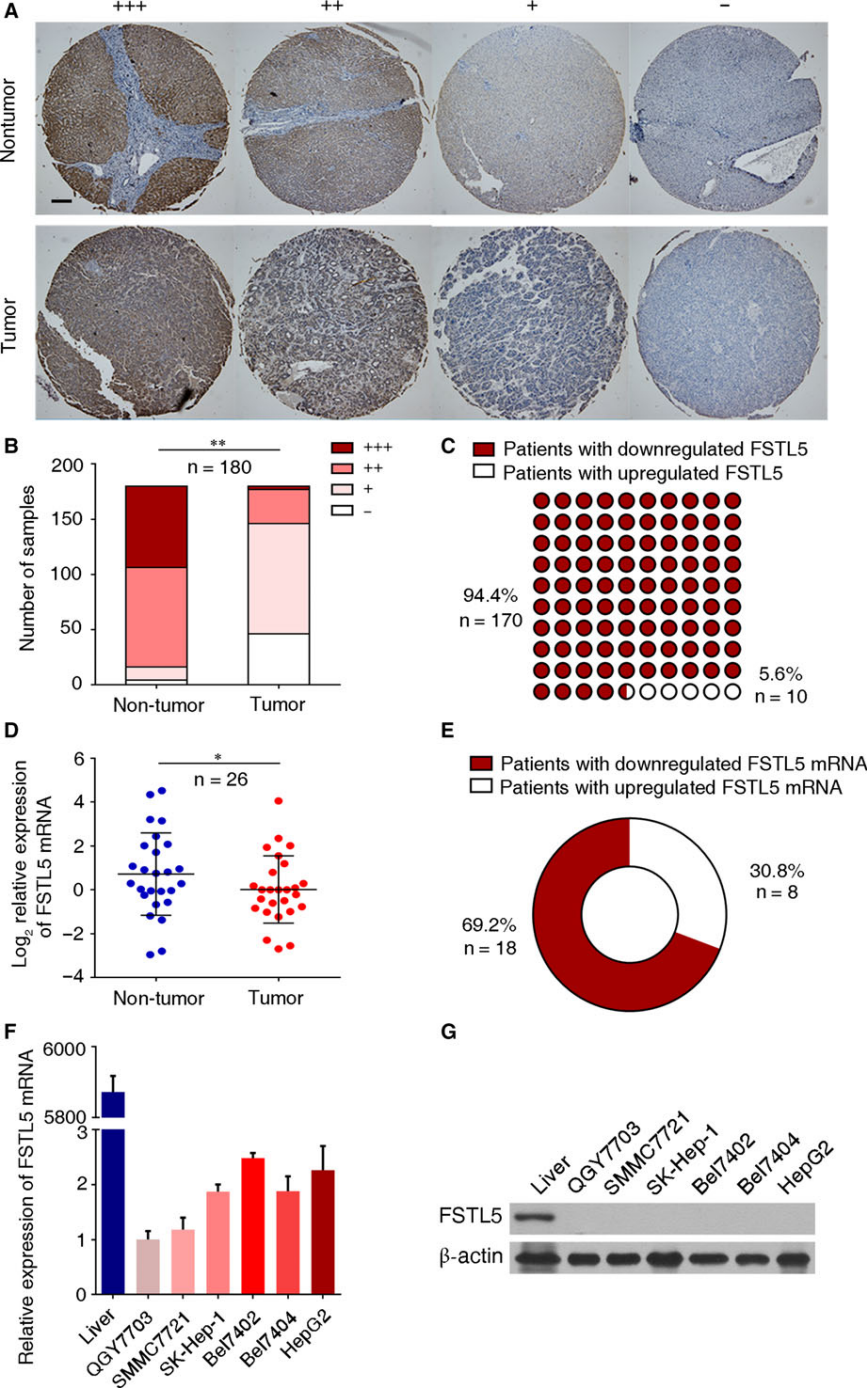
FSTL5 expression is downregulated in HCC tissues and cell lines. (A) Representative IHC staining of liver cancer tissue microarray (scale bar = 100 μm). (B) Number of samples with different IHC scores in both non‐tumor tissue and tumor tissue (n = 180, **P < 0.01, Student's *t* test). (C) Number of HCC samples with downregulated/upregulated FSTL5 (n = 180). (D) *FSTL5* mRNA expression in non‐tumor tissue and tumor tissue (n = 26, *P < 0.05, Student's *t* test). (E) Number of HCC samples with downregulated/upregulated *FSTL5* mRNA (n = 180). (F) qPCR showing *FSTL5* mRNA expression in HCC cell lines. (G) Western blotting showing FSTL5 protein expression in HCC cell lines

To investigate the expression of FSTL5 in liver cancer, IHC staining was performed to detect FSCT5 expression in a liver cancer TMA containing 180 samples. Then, we performed immunostaining grading analysis according to both the proportion of positively stained cells and the intensity of staining (Figure 1A). According to the IHC of the liver TMA, FSTL5 expression in tumor tissues was downregulated related to normal liver tissues (n = 180, **P < 0.01) (Figure 1B). The proportion of patients with HCC with downregulated FSTL5 expression in the tumor tissue was 94.4% (170/180 cases) (Figure 1C). Meanwhile, FSTL5 mRNA expression in HCC patient tissue obtained from West China Hospital was determined with qPCR and the result showed that FSTL5 mRNA expression in tumor tissues was also downregulated (n = 26, *P < 0.05) (Figure 1D). Out of 26 cases, the proportion of patients with HCC with downregulated expression of FSTL5 mRNA in the tumor tissue was 69.2% (18/26 cases) (Figure 1E). Then, the expression of FSTL5 mRNA and FSTL5 protein was detected in six HCC cell lines by qPCR and western blotting, respectively. Compared to normal human liver tissue, six HCC cell lines (QGY7703, SMMC7721, SK‐Hep‐1, Bel7402, Bel7404, and HepG2) barely expressed FSTL5 (Figure 1F and G). These findings indicated that FSTL5 is downregulated in HCC.

### 3.2 FSTL5 expression indicates favourable prognosis of patients with HCC at tumor node metastasis stage I/II

**Figure 2:**
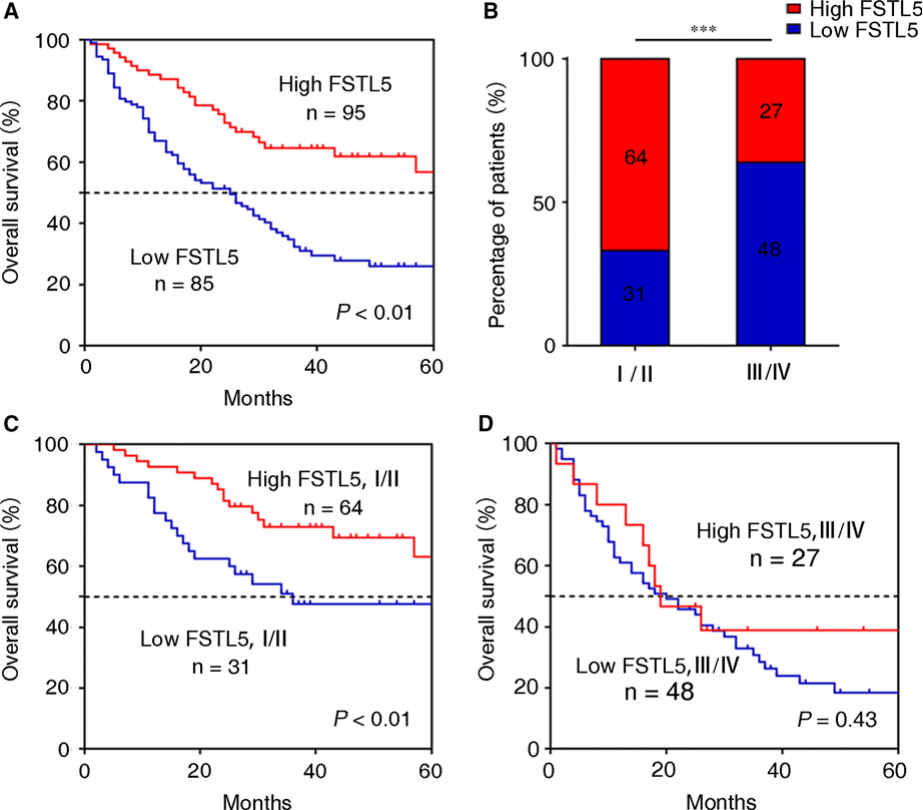
FSTL5 expression correlates with increased survival in patients with HCC at TNM stage I/II. (A) Kaplan–Meier curve showing overall survival of patients with HCC (n = 180, *P* < 0.01, log‐rank test). (B) Percentage of patients with high and low expression of FSTL5 at different TNM stages (n = 180, ***P < 0.001, Student's *t* test). (C) Kaplan–Meier curve showing overall survival of patients with HCC at TNM stage I/II (n = 105, *P* < 0.01, log‐rank test). (D) Kaplan–Meier curve showing overall survival of patients with HCC at TNM stage III/IV (n = 75, *P* = 0.43, log‐rank test)

To explore the clinical significance of FSTL5 in HCC, survival analysis of TMA was conducted, which showed that FSTL5 expression indicated favourable prognosis in patients with HCC (Figure 2A). Moreover, FSTL5 expression was associated with tumor node metastasis (TNM) stage in patients with HCC (Figure 2B). Therefore, we further studied the prognostic value of FSTL5 expression in patients with HCC at different TNM stages. As shown in Figure 2C, FSTL5 expression was positively correlated with prognosis of HCC at TNM stage I/II (n = 95, *P* < 0.01) (Figure 2C), and there was no significant correlation with the prognosis of patients with HCC at TNM stage III/IV (n = 75, *P* = 0.43) (Figure 2D). Then, we found that FSTL5 expression level had no significant correlation with gender (n = 180), age (n = 179), liver cirrhosis (n = 180), and tumor number (n = 179) in HCC (Table 1). Nevertheless, FSTL5 expression was significantly associated with tumor size (*P* = 0.0024), TNM stage (*P* < 0.0001), and histological grade (*P* = 0.0037) (Table 1). The univariate and multivariate analysis of factors associated with overall survival of patients with HCC showed that FSTL5 also can be used as an independent prognostic factor (*P* = 0.003) (Table 2). These findings suggested that FSTL5 could be an independent prognostic biomarker in HCC.

**Table 1:** Correlations of FSTL5 expression with clinical characteristics of HCC patients

Characteristics	Number of patients	FSTL5 expression		*P*‐value
		Low	High	
Gender (%)				
Male	152 (84.4)	72 (84.7)	80 (84.2)	0.9271
Female	28 (15.6)	13 (15.3)	15 (15.8)	
Age (%)				
≤60	133 (74.3)	64 (76.2)	69 (72.6)	0.5866
>60	46 (25.7)	20 (23.8)	26 (27.4)	
Cirrhosis (%)				
No	130 (72.2)	66 (77.6)	64 (67.4)	0.1243
Yes	50 (27.8)	19 (22.4)	31 (32.6)	
Tumor size (%)				
≤5 cm	79 (44.1)	27 (32.1)	52 (54.7)	**0.0024**
>5 cm	100 (55.9)	57 (67.9)	43 (45.3)	
Tumor number (%)				
=1	165 (92.2)	76 (90.5)	89 (93.7)	0.425
>1	14 (7.8)	8 (9.5)	6 (6.3)	
TNM stage (%)				
I/II	95 (55.9)	31 (39.2)	64 (70.3)	**<0.001**
>II	75 (44.1)	48 (60.8)	27 (29.7)	
Histological grade (%)				
I/II	121 (67.2)	48 (56.5)	73 (76.8)	**0.0037**
>II	59 (32.8)	37 (43.5)	22 (23.2)	

A *P*‐value <0.05 was considered to indicate statistical significance. The *P*‐values were calculated in IBM SPSS Statistics 22.0 using chi‐squared test.

**Table 2:** Univariate and multivariate analysis of factors associated with overall survival of HCC patients

Factors	Univariate analysis		Multivariate analysis	
	Hazard ratio (95% CI)	*P*‐value	Hazard ratio (95% CI)	*P*‐value
FSTL5	0.571 (0.424‐0.771)	**<0.001**	0.601 (0.429‐0.843)	**0.003**
Gender	0.937 (0.625‐1.405)	0.753		0.949
Age	0.970 (0.691‐1.362)	0.86		0.994
Cirrhosis	0.804 (0.577‐1.122)	0.2		0.638
Tumor size	1.673 (1.237‐2.263)	**0.001**		0.513
Tumor number	1.242 (0.718‐2.148)	0.439		0.761
TNM stage	1.720 (1.261‐2.346)	**0.001**	1.654 (1.176‐2.326)	**0.004**
Histological grade	1.102 (0.803‐1.512)	0.549		0.689

Hazard ratios (95% confidence interval; CI) and *P*‐values were calculated using univariate or multivariate Cox proportional hazards regression in IBM SPSS Statistics 22.0. A *P*‐value <0.05 was considered to indicate statistical significance.

### 3.3 FSTL5 suppresses HCC cell line growth by inducing apoptosis in vitro

**Figure 3:**
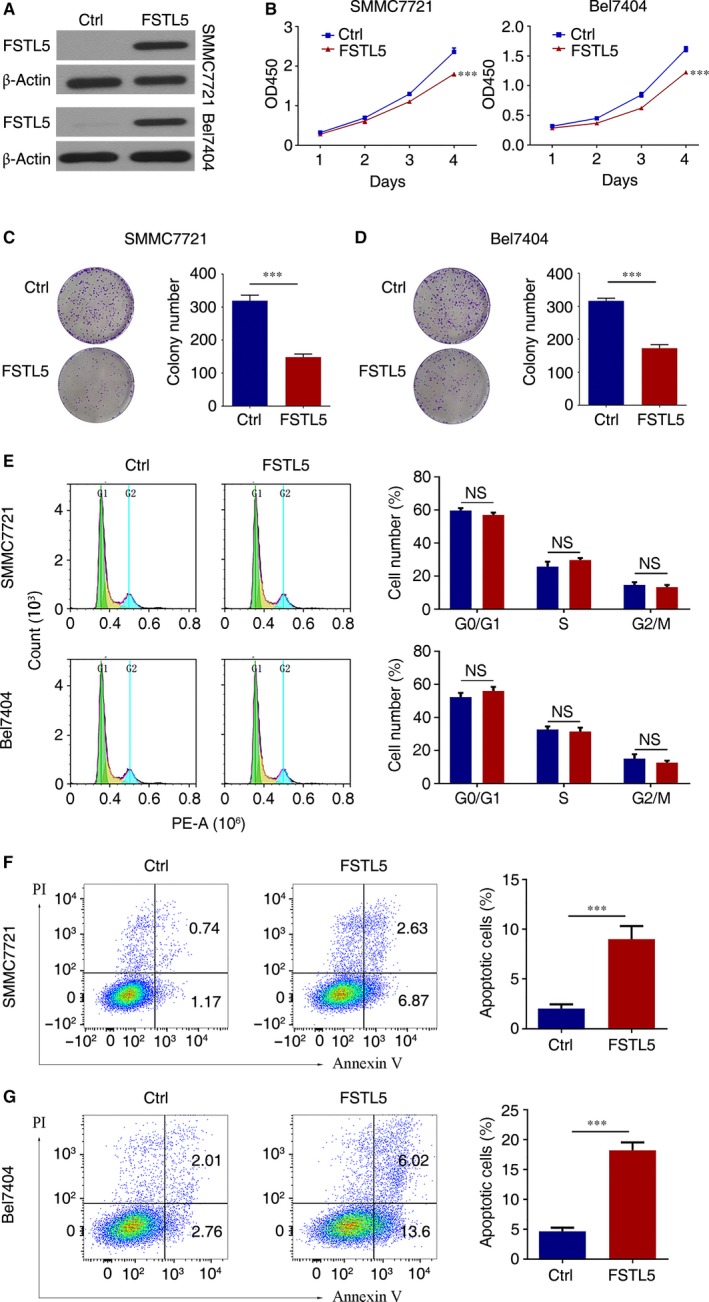
FSTL5 suppresses growth of HCC cell lines by inducing apoptosis in vitro. (A) Western blotting showing plasmid transfection efficiency. (B) CCK‐8 assay results showing the growth curve of SMMC7721/Bel7404 cells after transfection with pcDNA3.1(+)‐FSTL5 or pcDNA3.1(+) (n = 5, ***P < 0.001, Student's *t* test). (C and D) Colony formation assay of SMMC7721/Bel7404 cells after transfection with pcDNA3.1(+)‐FSTL5 or pcDNA3.1(+) (n = 3, ***P < 0.001, Student's *t* test). (E) Flow cytometry assay to determine cell cycle stages of SMMC7721/Bel7404 cells 72 hours after transfection with pcDNA3.1(+)‐FSTL5 or pcDNA3.1(+) (n = 3, NS, Student's *t* test). (F and G) Flow cytometry assay for apoptosis of SMMC7721/Bel7404 48 hours after transfection with pcDNA3.1(+)‐FSTL5 or pcDNA3.1(+) (n = 3, ***P < 0.001, Student's *t* test)

To determine the functional role of FSTL5 in regulating HCC growth in vitro, we transfected SMMC7721 and Bel7404 cells with an FSTL5 overexpression plasmid, followed by CCK‐8 and colony formation assays. Western blotting confirmed the ectopic expression of FSTL5 in FSTL5 overexpression plasmid transfected SMMC7721 and Bel7404 cells (Figure 3A). CCK‐8 results showed that, compared with the control group, FSTL5 overexpression inhibited the growth of SMMC7721 and Bel7404 cells (n = 6, ***P < 0.001, Student's *t* test) (Figure 3B). The colony formation assay yielded similar results; FSTL5 overexpression suppressed the colony formation ability of SMMC7721 and Bel7404 cells (n = 3, ***P < 0.001, Student's *t* test) (Figure 3C and D). CCK8 and colony formation results suggested that FSTL5 inhibited HCC growth in vitro.

To further validate the mechanism by which FSTL5 inhibits HCC growth, we assessed the effect of FSTL5 overexpression on cell cycle, and apoptosis. Flow cytometry results showed that FSTL5 overexpression in SMMC7721 and Bel7404 had no significant effect on the cell cycle (n = 3) (Figure 3E). Forty‐eight hours after overexpressing FSTL5 in SMMC7721 and Bel7404 cells, Annexin V‐FITC/PI double staining was conducted. Flow cytometry results showed that in SMMC7721 cells, the apoptotic cells in the control group were only 1.91%, while the apoptotic cells in the FSTL5 group increased up to 9.5% (n = 3, ***P < 0.001, Student's *t* test) (Figure 3F). In Bel7404 cells, the apoptotic cells in the control group was 4.77%, while the apoptotic cells in the FSTL5 group increased up to 19.62% (n = 3, ***P < 0.001, Student's *t* test) (Figure 3G). The above results clarified that FSTL5 inhibits HCC growth by promoting HCC cell apoptosis, instead of influencing cell cycle.

### 3.4 Knockdown of FSTL5 promotes HCC cell line and LO2 growth in vitro

**Figure 4:**
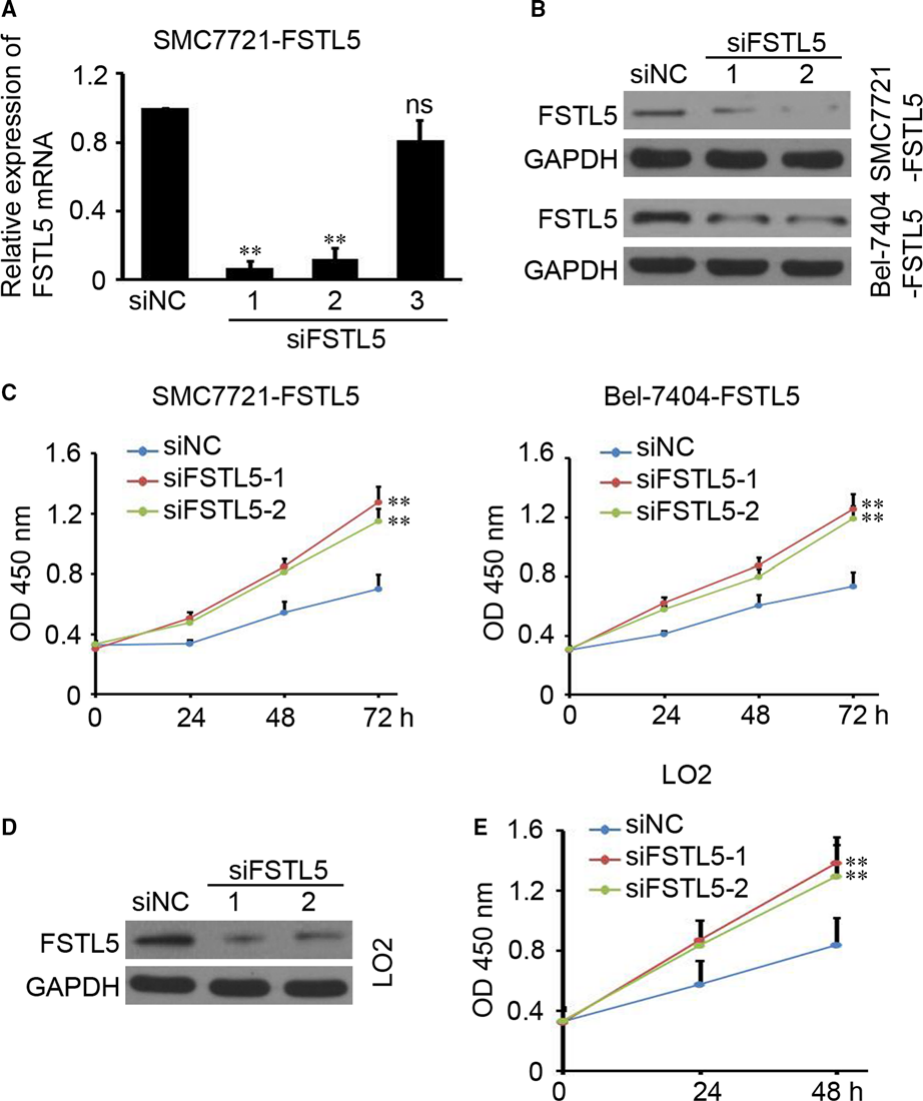
Knockdown of FSTL5 promotes HCC cell line and LO2 growth in vitro. (A) qPCR showing *FSTL5* mRNA expression in SMC7721‐FSTL5 cells after transfection siRNA targeting FSTL5 and siNC (negative control) (n = 4, **P < 0.01, Student's *t* test; ns, no significant difference). (B) Western blotting analysis of FSTL5 protein expression in SMC7721‐FSTL5 and Bel‐7404‐FSTL5 cells after transfection siRNA targeting FSTL5 and siNC. (C) CCK‐8 assay results showing the growth curve of SMC7721‐FSTL5 and Bel‐7404‐FSTL5 cells after transfection siRNA targeting FSTL5 and siNC (n = 5, **P < 0.01, Student's *t* test). (D) Western blotting analysis of FSTL5 protein expression in LO2 cells after transfection siRNA targeting FSTL5 and siNC. (E) CCK‐8 assay results showing the growth curve of LO2 cells after transfection siRNA targeting FSTL5 and siNC (n = 5, **P < 0.01, Student's *t* test)

Next, three siRNAs targeting FSTL5 were purchased and used to transfect FSTL5‐overexpressed SMC7721 cell (SMC7721‐FSTL5), and the results suggested that siFSTL5‐1 and siFSTL5‐2 efficiently knockdown FSTL5 expression in SMC7721‐FSTL5 cell (Figure 4A). Furthermore, western blotting results also confirmed the knockdown of FSTL5 protein by siFSTL5‐1 and siFSTL5‐2 both in SMC7721‐FSTL5, Bel‐7404‐FSTL5, and LO2 cell (Figure 4B and D). CCK‐8 assay indicated that knockdown of FSTL5 significantly promote SMC7721‐FSTL5, Bel‐7404‐FSTL5, and LO2 cell growth in vitro (Figure 4C and E). The above results indicated that knockdown of FSTL5 promotes HCC cell line and LO2 cell growth in vitro.

### 3.5 FSTL5 induces caspase‐dependent apoptosis through regulating Bcl‐2 family proteins in HCC

**Figure 5:**
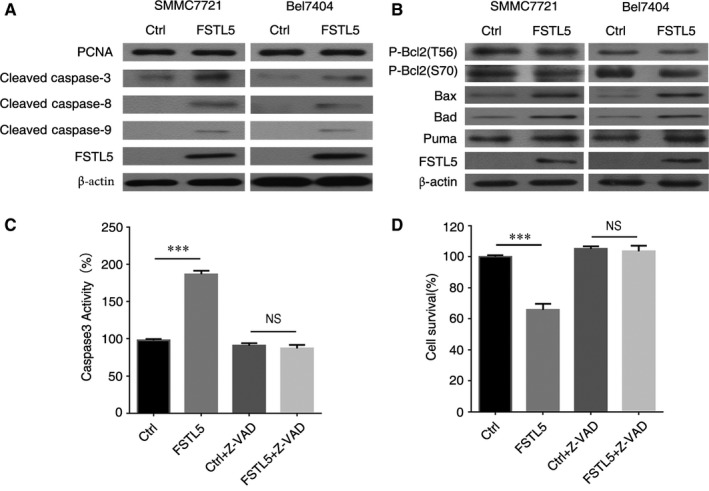
FSTL5 induces caspase‐dependent apoptosis and regulates Bcl‐2 family proteins in HCC. (A and B) Western blotting in SMMC7721/Bel7404 48 hours after transfection with pcDNA3.1(+)‐FSTL5 or pcDNA3.1(+). (C) SMMC7721 cells were transfected with pcDNA3.1(+)‐FSTL5 or pcDNA3.1(+), and then treated with 10 μM Z‐VAD‐FMK for 48 hours, and caspase‐3 activity was measured by a Caspase‐3 Activity Assay Kit (n = 3, ***P < 0.001, Student's *t* test). (D) SMMC7721 cells were transfected with pcDNA3.1(+)‐FSTL5 or pcDNA3.1(+), and then treated with 10 μM Z‐VAD‐FMK for 48 hours, and cell survival was measured by CCK‐8 assay (n = 3, ***P < 0.001, Student's *t* test)

We explored the mechanism of how FSTL5 induces apoptosis in HCC. Forty‐eight hours after transfection with FSTL5 overexpression plasmid, levels of Cleaved Caspase‐3, ‐8, and ‐9 were increased in SMMC7721 and Bel7404 cells (Figure 5A), whereas has no significant effect on the expression of PCNA (Figure 5A). Furthermore, Bcl‐2 family proteins including pro‐apoptotic proteins Bax, Bad, and Puma, and the anti‐apoptotic protein Bcl‐2 were also investigated. FSTL5 downregulated the levels of the anti‐apoptotic protein P‐Bcl2(T56) and P‐Bcl2(S70), and upregulated the expression of the pro‐apoptotic proteins Bax, Bad, and Puma in the mitochondrial pathway (Figure 5B). The caspase inhibitor Z‐VAD‐FMK effectively reversed the elevated Caspase‐3 activity caused by FSTL5 overexpression in SMMC7721 cells and prevented FSTL5‐induced apoptosis (Figure 5C and D). The above results showed that FSTL5 promoted cell apoptosis in a caspase‐dependent manner through regulating Bcl‐2 family protein in HCC.

### 3.6 FSTL5 recombinant protein promotes apoptosis and regulates Bcl‐2 family proteins in a dose‐dependent manner in HCC

**Figure 6:**
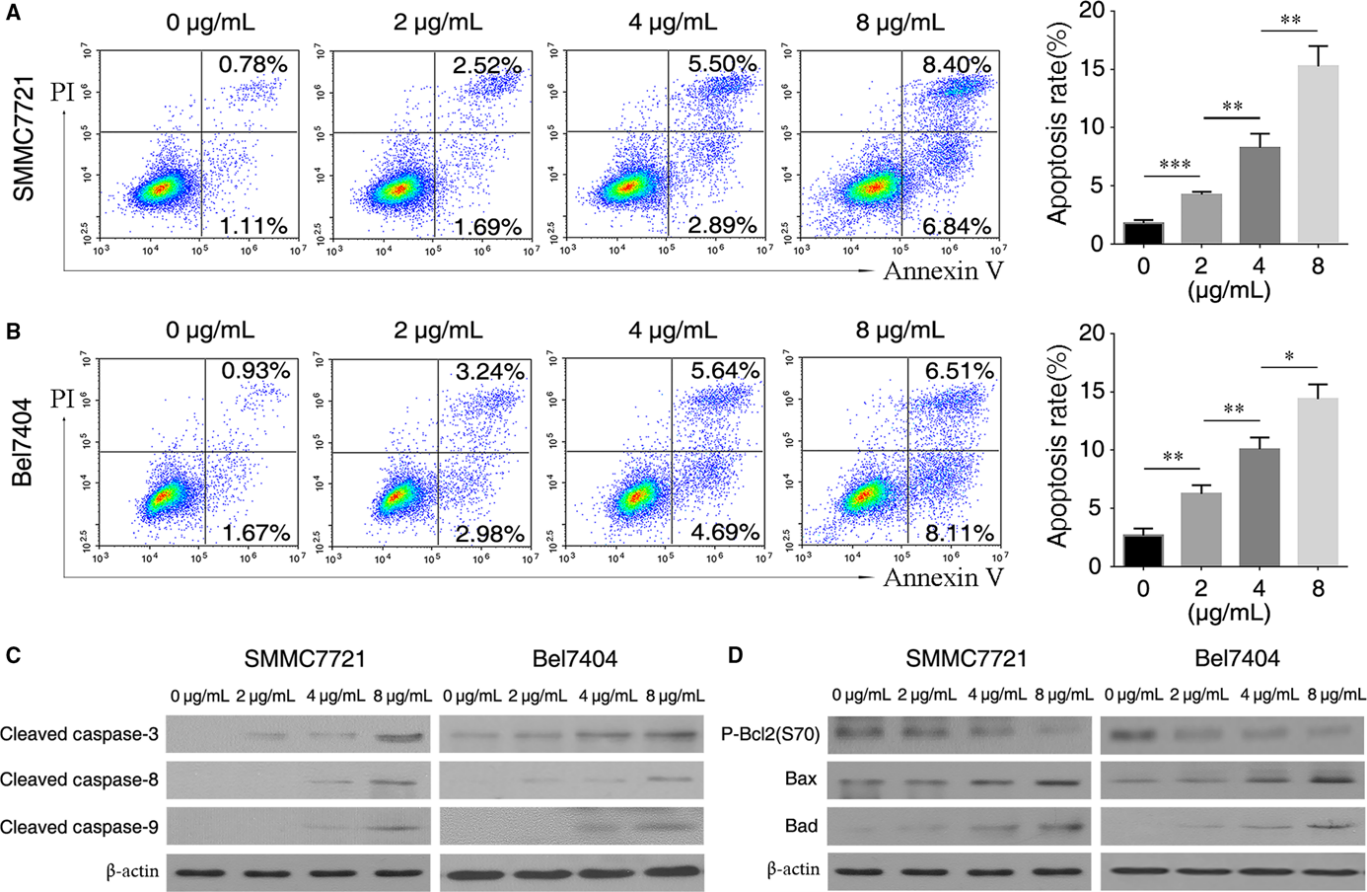
FSTL5 recombinant protein promotes apoptosis and regulates Bcl‐2 family proteins in a dose‐dependent manner in HCC. (A and B) Flow cytometry assay for apoptosis of SMMC7721/Bel7404 cells 48 hours after treatment with FSTL5 recombinant protein at various doses, *P < 0.05, **P < 0.01, ***P < 0.001, Student's *t* test). (C and D) Western blotting analysis of SMMC7721/Bel7404 cells 48 hours after treatment with FSTL5 recombinant protein at various doses

FSTL5 was demonstrated as a secreted protein.^20^ Thus, recombinant protein was used to treat the HCC cells and determine whether FSCTL5 induce HCC cells apoptosis with a exogenous manner. As shown in Figure 5A and B, in the presence of recombinant FSTL5 protein at doses of 0, 2, 4, and 8 μg/mL, the apoptosis rates of SMMC7721 cells were 1.89%, 4.21%, 8.39%, and 15.24% respectively, and apoptosis rates of Bel7404 cells were 2.6%, 6.22%, 10.33%, and 14.66%, respectively (n = 3, *P < 0.05, **P < 0.01, ***P < 0.001, Student's *t* test) (Figure 6A and B). Western blotting showed that different doses of FSTL5 recombinant protein increased the levels of Cleaved Caspase‐3, ‐8, ‐9, Bax, and Bad, meanwhile decreasing P‐Bcl2(S70) levels to varying degrees (Figure 6C and D). The above results showed that the FSTL5 recombinant protein promoted HCC cell apoptosis through regulating the caspase pathway and Bcl‐2 family protein in a dose‐dependent manner.

### 3.7 FSTL5 inhibits HCC tumor growth in vivo

**Figure 7:**
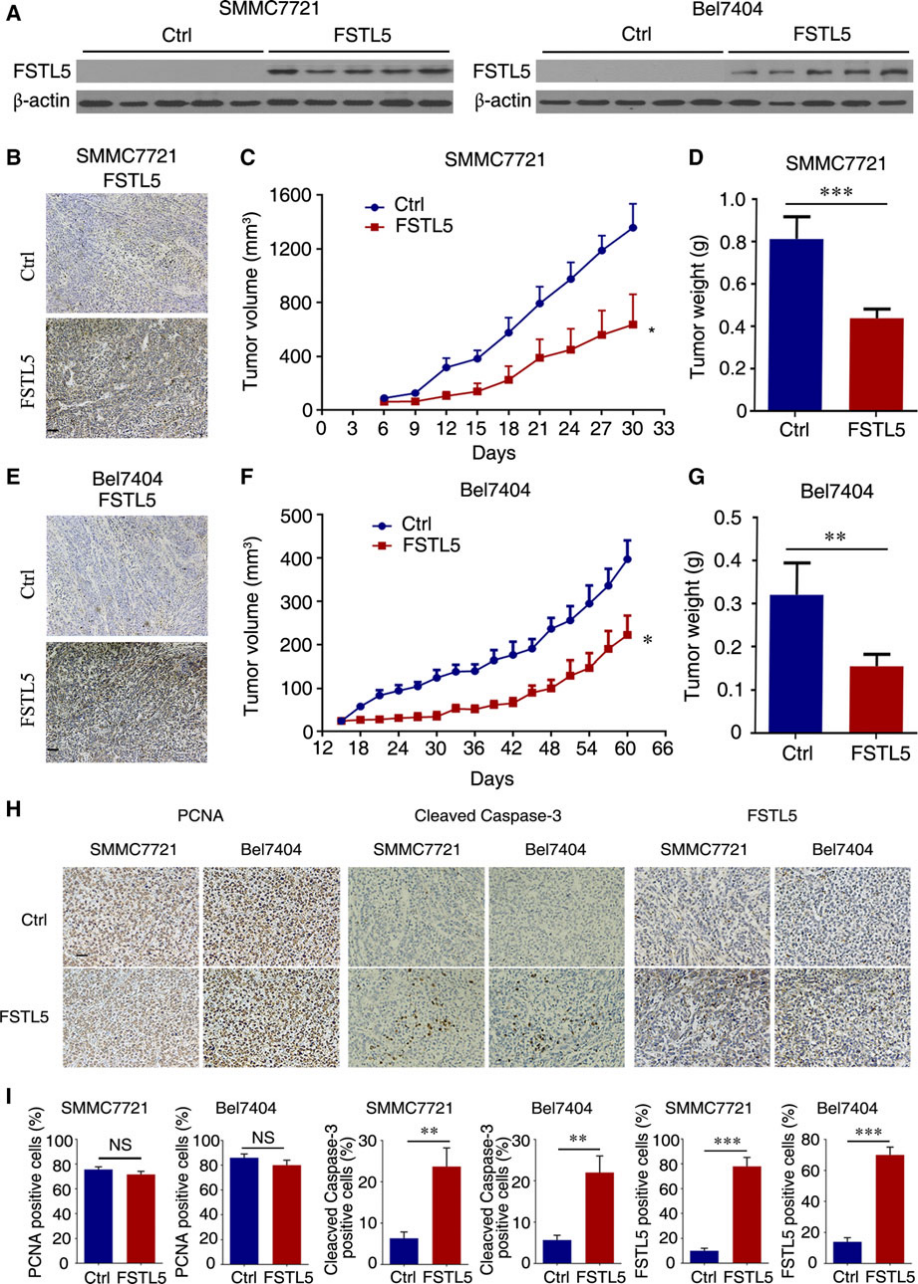
FSTL5 inhibits HCC tumor growth in vivo. (A) Western blotting showing FSTL5 expression in FSTL5 and control SMMC7721/Bel7404 xenografts. (B and E) IHC staining for FSTL5 in FSTL5‐expressing and control SMMC7721/Bel7404 xenografts (scale bar = 50 μm). (C and D) Tumor volume (n = 5, *P < 0.05, Student's *t* test), and end‐stage tumor weight (n = 5, ***P < 0.001, Student's *t* test), after injection of FSTL5‐expressing and control SMMC7721 cancer cells into nude mice. (F and G) Tumor volume (n = 5, *P < 0.05, Student's *t* test), and end‐stage tumor weight (n = 5, **P < 0.01, Student's *t* test), after injection of FSTL5‐expressing and control Bel7404 cancer cells into nude mice. (H and I) IHC staining for PCNA, Cleaved Caspase‐3 and FSTL5 in xenografts from nude mice (n = 3, **P < 0.01, ***P < 0.001, Student's *t* test, scale bar = 25 μm)

To examine whether FSTL5 expression affected HCC growth in vivo, we employed a gain‐of‐function approach to study FSTL5 function in SMMC7721 and Bel7404 cells. The cells were infected with LV containing either empty vector (LV‐Ctrl) or a vector expressing human FSTL5 (LV‐FSTL5). Cells stably expressing the constructs were selected by puromycin and named SMMC7721‐control, SMMC7721‐FSTL5, Bel7404‐control, and Bel7404‐FSTL5. Western blotting, and IHC showed that FSTL5 was efficiently expressed in SMMC7721‐FSTL5 and Bel7404‐FSTL5 cells, but not in control cells (Figure 7A, B and E). FSTL5 overexpression remarkably decreased tumor volume and weight of SMMC7721 cell xenografts by 53.1% and 46%, respectively; tumor volume: control: 1357 ± 434.9 mm^3^ vs FSTL5: 636.6 ± 102.7 mm^3^; and tumor weight: control: 0.815 ± 0.104 g vs FSTL5: 0.44 ± 0.04 g) (Figure 7C and D). FSTL5 overexpression also decreased tumor volume and weight of Bel7404 cell xenografts by 44.1% and 51.4%, respectively; tumor volume: control: 397.2 ± 106.1 mm^3^ vs FSTL5: 222 ± 109.7 mm^3^; and tumor weight: control: 0.321 ± 0.074 g vs FSTL5: 0.156 ± 0.026 g) (Figure 7F and G). Subcutaneous tumor paraffin section IHC results showed that PCNA staining in the FSTL5 group and control group were not significantly different (n = 3), and FSTL5 upregulated levels of Cleaved Caspase‐3 (Figure 7H and I). These results indicated that FSTL5 inhibited HCC tumor growth in vivo through promoting apoptosis.

## 4 DISCUSSION

FSTL family members show altered expression in cancer. FSTL1 was found to be downregulated in nasopharyngeal cancer^22^ but upregulated in primary glioma.^23^ The expression of FSTL3 in endometrial cancer is decreased at early stages, and increased in tumor capillaries.^24^ Furthermore, FSTL3 was elevated in the liver cancer,^25^ in invasive breast cancer.^26^, ^27^ Whereas, in patients with endometriosis and ovarian cancer, FSTL3 mRNA expression is downregulated compared with levels observed in healthy people.^28^ FSTL5 DNA copy number and protein was demonstrated to be reduced in most patients with HCC.^19^, ^20^ In our study, we first determined the FSTL5 mRNA expression in HCC tissues and confirmed the consistency of FSTL5 mRNA and protein expression (downregulated) in HCC and HCC cell lines. Furthermore, the clinical significance of FSTL5 expression in patients with HCC was also investigated and suggested that FSTL5 was positively correlated with favourable prognosis in patients with liver cancer, which is consistent with prior study by Zhang et al^20^ Of note, our results firstly demonstrated that FSTL5 expression indicated favourable prognosis of patients with HCC at TNM stage I/II, while it had no significant correlation with the prognosis of patients with HCC at TNM stage III/IV. This result may provide deep profound result for understanding the clinical significance of FSTL5 in HCC. But further investigations are needed to explain the opposite results between our study and previous study by Remke et al, which found that FSTL5 was positively correlated with the adverse prognosis of non‐WNT/non‐SHH medulloblastoma.^17^

Zhang et al found that FSTL5 could promote HCC cell apoptosis by affecting WNT/β‐catenin signalling in vitro.^20^ In our study, we first studied the effect of FSTL5 on HCC growth both in vivo and in vitro and found that overexpressing FSTL5 could effectively inhibit the growth of HCC cells and HCC xenografts. Tumor growth is a complex biological process, and its main influencing factors are cell cycle, and apoptosis.^29^ Therefore, to explore the mechanism of FSTL5 in inhibiting growth of HCC, we determined the effects of FSTL5 on cell cycle and apoptosis of HCC in vitro. FSTL5 has no significant effect on cell cycle of HCC either in vivo or in vitro, evidenced by flow cytometry analysis and PCNA measuring. Meanwhile, our study showed that FSTL5 expression can effectively promote the apoptosis of HCC cells and liver tumors. This is the first study to demonstrate that FSTL5 could inhibit the xenograft tumor growth of liver cancer by promoting HCC apoptosis, rather than influencing cell cycle.

Excessive growth caused by cells evading apoptosis is an important cause of tumor occurrence.^29^ Cell apoptosis mainly includes the extrinsic death receptor pathway and intrinsic mitochondrial pathway.^30^ Extrinsic death receptor pathways are usually triggered by ligands of the TNF family's death receptors, including TNF, Fas ligands, and TRAIL.^31^ The intrinsic mitochondrial pathway is usually regulated by Bcl2 family members, which leads to Caspase pathway activation by controlling mitochondrial membrane permeability and cytochrome c release following activation of Caspase‐9.^32^, ^33^, ^34^, ^35^ Both initiator caspases of the intrinsic and extrinsic result in their own autoactivation which further activates Caspase‐3, the effector caspase.^36^ Here, we studied the downstream signalling pathway of apoptosis. The overexpression of FSTL5 increased the levels of Cleaved Caspase‐3, ‐8, and ‐9. Since FSTL5 can effectively activate important proteins in the caspase pathway, we speculated that FSTL5‐mediated apoptosis may be caspase‐dependent. Therefore, we used the pan caspase inhibitor Z‐VAD‐FMK to treat SMMC7721 cells overexpressing FSTL5 and found that Z‐VAD‐FMK could inhibit cell FSTL5‐induced apoptosis. These results suggested that FSTL5‐mediated cancer cell apoptosis occurs in a caspase‐dependent manner. However, whether and how the intrinsic and extrinsic apoptosis pathways are involved in this process still needs to be determined in our future study.

Among the Bcl‐2 family members, the balance between pro‐apoptotic and anti‐apoptotic members determines cell fate.^37^ Bcl‐2 responds to a series of apoptotic factors by inhibiting the release of mitochondrial cytochrome c, which can promote cell survival.^38^ In the process of apoptosis induced by T lymphocyte glucocorticoids, Bcl‐2 mutation at Thr56 or Ser87 can inhibit its anti‐apoptotic activity.^39^ The phosphorylation of Bcl‐2 induced by interleukin 3 and JNK at Ser70 site is necessary to enhance anti‐apoptosis activity.^40^ Bax and Bad are important component of the process of inducing intrinsic apoptosis.^41^, ^42^ Puma can bind to anti‐apoptotic Bcl‐2 family members to induce mitochondrial dysfunction and caspase activation.^43^, ^44^, ^45^, ^46^ We found that FSTL5 lowered levels of the anti‐apoptotic proteins P‐Bcl2(T56) and P‐Bcl2(S70) and increased levels of pro‐apoptotic proteins Bax, Bad, and Puma with a exogenous manner. Nevertheless, the mechanism underlying FSTL5 regulating Bcl‐2 family proteins requires further study.

In summary, we found that FSTL5 was downregulated in HCC cell and HCC tissue and FSTL5 expression positively correlated with good prognosis in patients with HCC at TNM stages I/II rather than TNM stage III/IV. The results in vitro and in vivo show that the FSTL5 inhibit HCC growth by promoting caspase‐dependent apoptosis of HCC cells and regulating Bcl‐2 family proteins in HCC, rather than influencing cell cycle. Our study suggested that FSTL5 is a potential target and molecular marker for the diagnosis, treatment, and prognosis predicting in HCC. However, a more detailed molecular mechanism remains to be elucidated in the further study.

### 4.1 Ethics approval and consent to participate

All procedures were undertaken in accordance with the Declaration of Helsinki. The collection and use of the samples were reviewed and approved by the Institutional Ethics Committee of the West China Hospital of Sichuan University (China).

## CONFLICT OF INTEREST

The authors confirm that there are no conflicts of interest.

## AUTHOR CONTRIBUTION

HXD and CLL conceived the project and supervised research. LD, JFZ, YJZ, and YW drafted the manuscript. YL, LC, and HWT contributed the original data. CLL, XZ, QNW, and QMY designed and carried out the experiments. GS, FYC, and XLS collected the clinical samples. YY and SZ were involved in statistical analysis. DCY and YQW provided essential reagents or tools. All authors read and approved the final version of the manuscript.
