# Corrigendum

**DOI:** 10.1111/jcmm.16364

**Published:** 2021-04-09

In Chunlei Li et al., the published article contains errors in Figure 6D, Figure 7A and Figure 7H. The correct figures are shown below. The authors confirm all results, and conclusions of this article remain unchanged.

**FIGURE 6:**
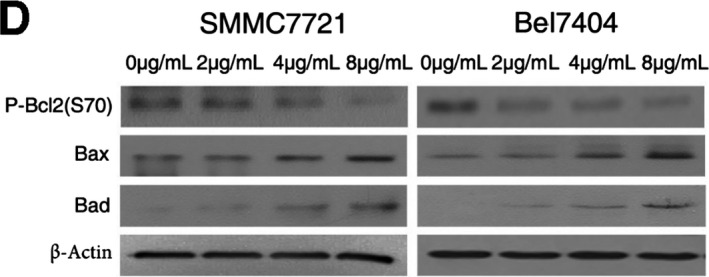
FSTL5 recombinant protein promotes apoptosis and regulates Bcl‐2 family proteins in a dose‐dependent manner in HCC. (A and B) Flow cytometry assay for apoptosis of SMMC7721/Bel7404 cells 48 h after treatment with FSTL5 recombinant protein at various doses, *P < .05, **P < .01, ***P < .001, Student's *t* test). (C and D) Western blotting analysis of SMMC7721/Bel7404 cells 48 h after treatment with FSTL5 recombinant protein at various doses

**FIGURE 7:**
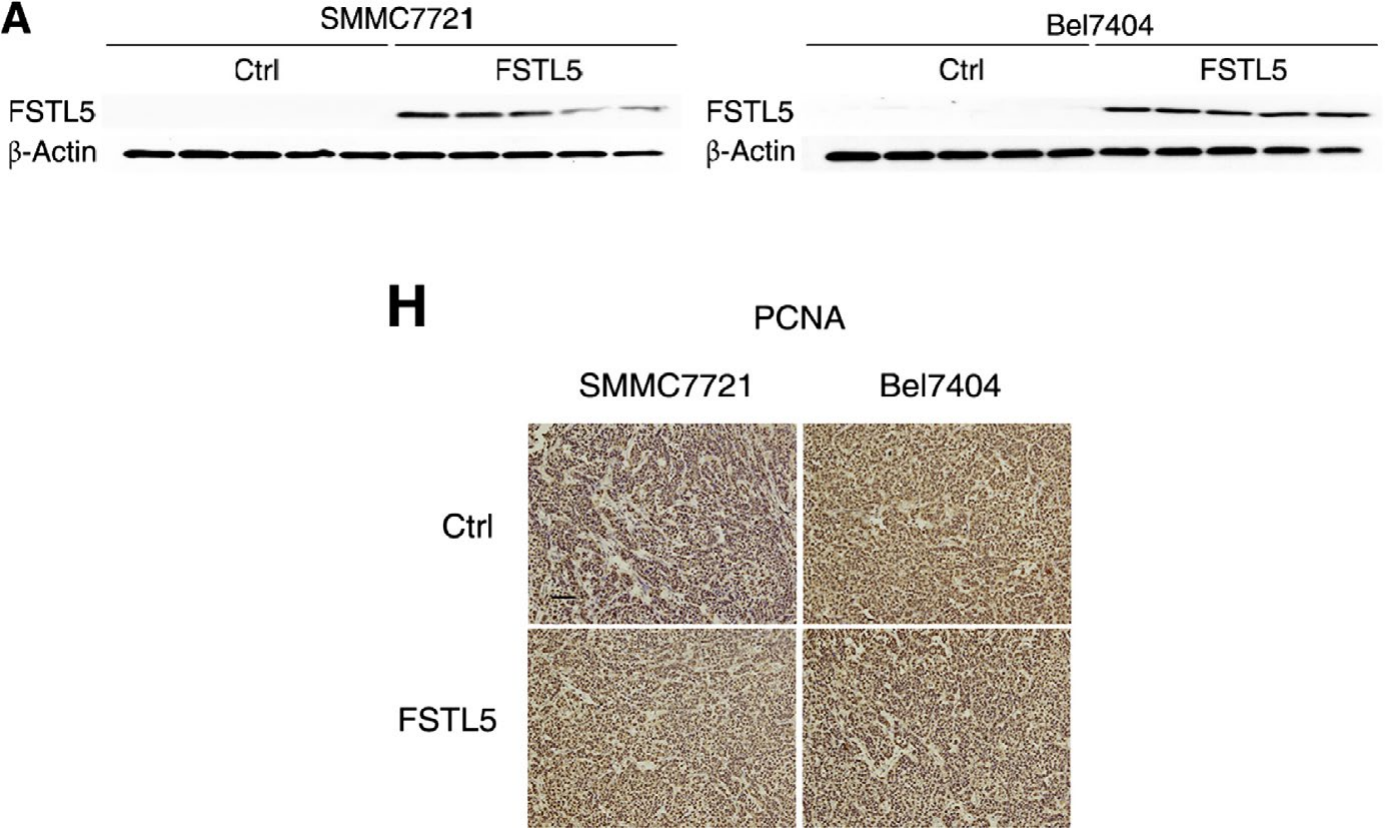
FSTL5 inhibits HCC tumour growth in vivo. (A) Western blotting showing FSTL5 expression in FSTL5 and control SMMC7721/Bel7404 xenografts. (B and E) IHC staining for FSTL5 in FSTL5‐expressing and control SMMC7721/Bel7404 xenografts (scale bar = 50 μm). (C and D) Tumour volume (n = 5, *P < .05, Student's *t* test) and end‐stage tumour weight (n = 5, ***P < .001, Student's *t* test), after injection of FSTL5‐expressing and control SMMC7721 cancer cells into nude mice. (F and G) Tumour volume (n = 5, *P < .05, Student's *t* test), and end‐stage tumour weight (n = 5, **P < .01, Student's *t* test), after injection of FSTL5‐expressing and control Bel7404 cancer cells into nude mice. (H and I) IHC staining for PCNA, cleaved caspase‐3 and FSTL5 in xenografts from nude mice (n = 3, **P < .01, ***P < .001, Student's *t* test, scale bar = 25 μm)
